# Dynamic Process of Secondary Pulmonary Infection in Mice With Intracerebral Hemorrhage

**DOI:** 10.3389/fimmu.2021.767155

**Published:** 2021-11-19

**Authors:** Hanyu Zhang, Yingying Huang, Xiaojin Li, Xu Han, Jing Hu, Bin Wang, Lin Zhang, Pengwei Zhuang, Yanjun Zhang

**Affiliations:** ^1^ College of Chinese Materia Medica, Tianjin University of Traditional Chinese Medicine, Tianjin, China; ^2^ Tianjin State Key Laboratory of Modern Chinese Medicine, Tianjin University of Traditional Chinese Medicine, Tianjin, China; ^3^ College of Pharmacy, Anhui University of Chinese Medicine and Anhui Academy of Chinese Medicine, Hefei, China

**Keywords:** intracerebral hemorrhage, immunosuppression, intestinal barrier dysfunction, translocation of intestinal flora, pulmonary infection

## Abstract

Stroke is a common central nervous system disease in clinical practice. Stroke patients often have infectious complications, such as pneumonia and infections of the urinary tract and gastrointestinal tract. Although it has been shown that translocation of the host gut microbiota to the lungs and immune dysfunction plays a vital role in the development of infection after ischemic stroke, the occurrence and mechanism of pulmonary infection at different time points after hemorrhagic cerebral remain unclear. In this study, the changes in the immune system and intestinal barrier function in mice during disease development were investigated at 1 day (M 1 d), 3 days (M 3 d) and 7 days (M 7 d) following hemorrhagic stroke to clarify the mechanism of secondary pulmonary infection. The experimental results revealed that after hemorrhagic stroke, model mice showed increased brain damage from day 1 to 3, followed by a trend of brain recovery from day 3 to 7 . After hemorrhagic stroke, the immune system was disturbed in model mice. Significant immunosuppression of the peripheral immune system was observed in the M 3 d group but improved in the M 7 d group. Staining of lung tissues with hematoxylin and eosin (H&E) and for inflammatory factors revealed considerable disease and immune disorders in the M 7 d group. Stroke seriously impaired intestinal barrier function in mice and significantly changed the small intestine structure. From 1 to 7 d after stroke, intestinal permeability was increased, whereas the levels of markers for intestinal tight junctions, mucus and immunoglobulin A were decreased. Analysis based on 16S rRNA suggested that the microflora in the lung and ileum was significantly altered after stroke. The composition of microflora in lung and ileum tissue was similar in the M 7d group, suggesting that intestinal bacteria had migrated to lung tissue and caused lung infection at this time point after hemorrhagic stroke. In stroke mice, the aggravation of intestinal barrier dysfunction and immune disorders after intracerebral hemorrhage, promoted the migration of enteric bacteria, and increased the risk of pneumonia poststroke. Our findings reveal the dynamic process of infection after hemorrhagic stroke and provide clues for the optimal timing of intervention for secondary pulmonary infection in stroke patients.

## 1 Introduction

Stroke is a common clinically acute cerebrovascular disease categorized as ischemic stroke and hemorrhagic stroke according to pathogenesis. Stroke is characterized by high disability and mortality. Although there have been many studies on hemorrhagic stroke (intracerebral hemorrhage, ICH), research on this topic lags significantly behind that on ischemic stroke. Infection is common after stroke and is associated with worse stroke outcomes (1). Approximately 23-58% of patients with ICH have infectious complications, including pneumonia (28%) and urinary tract infections (24%) (2). Poststroke infectious complications were reported to be a major reason for readmission and death of stroke patients and associated with poor outcomes (3–5). Moreover, treatment with the β-adrenergic receptor blocker propranolol did not reduce the risk of stroke-associated pneumonia (SAP) and had no direct effect on mortality within 7 d of stroke onset (6, 7). In fact, the optimal timing of treatment for lung infection remains undefined.

At present, effective prevention and treatment strategies for lung infection after stroke injury are still limited and contradictory. Previous experimental studies demonstrated that antibiotic prophylaxis reduced poststroke infection and improved other clinical outcomes (8). However, some clinical trials showed that antibiotic prophylaxis in patients with acute stroke did not reduce the incidence of poststroke pneumonia and was not associated with reduced mortality (9, 10). Therefore, it is essential to identify the processes underlying stroke-induced pulmonary infection to reduce infection-related mortality and improve rehabilitation after stroke.

The mechanisms underlying lung infection after a stroke are not fully understood. Clinical study reports have speculated that risk factors for poststroke pneumonia include aspiration, dysphagia, nasogastric tubing and mechanical ventilation (11). The consequences of the immune response during stroke are complicated. Both a proinflammatory response to stroke, or response to immunosuppression after stroke have been reported (12). Furthermore, stroke-induced immunosuppression is an important factor that predisposes the host to lung infections (5). Impaired consciousness and immunosuppression are critical in poststroke infection, as is the translocation of intestinal flora (13). Many researchers have used the rodent stroke model to study the role of intestinal flora in lung infection after stroke (13, 14). *Stanley et al.* reported the migration of intestinal bacteria from the host gut to lung tissue after stroke (13). Aged mice were found to be particularly more susceptible to immune dysfunction and infection than were younger mice (14, 15). Despite these findings, the timing of changes related to pulmonary infection in ICH has not been reported.

Given this background, an animal model of hemorrhagic stroke was used to study changes in the immune system (peripheral and local to the lung) and intestinal barrier function at the 1,3 and 7 d after stroke and to explore the possible mechanism of secondary pulmonary infection after hemorrhagic stroke. More data on the dynamic changes involved in secondary pulmonary infection processes in mice with ICH may be helpful to determine the optimal timing of treatment to prevent infection in the future.

## 2 Materials and Methods

### 2.1 Mice

C57BL/6 mice (6-8 weeks old, male, weighing 20 g) were supplied by the Animal Research Center of Tianjin University of Traditional Chinese Medicine. Mice were housed in a specific-pathogen-free (SPF) environment (temperature, 22 ± 1°C; relative humidity, 50 ± 1%; and normal day/night cycle, 12/12 h). The animals were randomly allocated to four groups (n=10 per group): (i) sham group, (ii) 1 d post stroke (M 1 d) group, (iii) 3 d post stroke (M 3 d) group, and (iv) 7 d post stroke (M 7 d) group. All animal experiments were performed in compliance with the requirements of the Animal Research Center of Tianjin University of Traditional Chinese Medicine (TCM-LAEC2019079).

### 2.2 Surgical Procedures

The mouse ICH model was induced by 0.03 U collagenase. All mice were anesthetized by an intraperitoneal injection of 10% chloral hydrate and placed on a brain stereotaxic apparatus (RWD, China) in the prone position. We injected 0.5 µL collagenase (0.06 U per 1 µL, Type IV-S; Sigma-Aldrich, St. Louis, MO, USA) at a coordinate point (0.5 mm anterior, 2.3 mm lateral, and 3.7 mm depth to bregma). The needle was kept at the injection point for 10 minutes after injection to prevent fluid reflux. Finally, the microsyringe was slowly removed, the skull pinhole was closed with bone wax, and the skin incision was closed. Mice in the sham group were injected with normal saline.

### 2.3 Measurement of the Brain Hematoma Area Ratio

The mouse brains were serially sectioned at 1-mm intervals anterior and posterior to the needle entry point as the datum plane. The total hematoma area(mm^2^) of each mouse brain was quantified by ImageJ software. The brain hematoma area ratio was calculated using the following formula: the brain hematoma area ratio was calculated by dividing the hematoma area by the brain slice area and multiplying by 100.

### 2.4 Measurement of the Brain Index

The brain index was measured in mouse cerebral tissues after ICH. Briefly, mice randomly sampled from each group were anesthetized by an intraperitoneal injection of chloral hydrate (n = 5). Next, cerebral tissues were removed, and the surface water on cerebral tissues was removed by blotting with filter paper. Brain samples were immediately weighed on an electric analytic balance to obtain the brain weight. The brain index was calculated as the ratio of brain weight (mg) to body weight (g).

### 2.5 Evaluation of the Neurobehavioral Score

Neurobehavioral scores were determined for experimental mice before sampling, and all tests were completed with the help of two students who were blinded to the experimental group. The test items are described below:

#### 2.5.1 Wire Hang Test 

A 50-cm long wire was placed between two platforms, located 40 cm away from the desktop. Mice were placed in the middle of the wire and scored according to their behavior: reached the platform or moved >25 cm in 60 s (5 points); used the front and rear limbs or tail to hold on to the wire for more than 30 s, moved ≤25 cm, and did not reach the platform within 60 s (4 points); held the paw symmetrically for more than 30 s, moved ≤25 cm, and failing to reach the platform within 60 s (3 points); adhered in any way to the wire for more than 30 s (2 points); clung to the wire for 15–30 s (1 point); and dropped off of the wire within 15 s (0 points). The test was repeated three times and the average of the three tests was calculated as previously described (16).

#### 2.5.2 Beam Balance Test

A 50-cm round stick was placed between two platforms located 40 cm away from the tabletop. Mice were placed in the middle of the bars and rated according to their behavior: reached the platform or moved more than 25 cm in ≤25 s (5 points); reached the platform or move more than 25 cm in ≤40 s (4 points); moved more than 10 cm≥25 s (3 points); moved less than 10 cm in ≥25 s (2 points); held still for ≥25 s (1 point); and dropped off the stick within 10 s (0 points). The test was repeated three times and the average of the three tests was calculated as previously described (17, 18).

#### 2.5.3 Symmetry of Limb Movement (Ls)

The mouse was suspended by the tail to evaluate limb movement, which was graded as follows: symmetrical limb extension (3 points); asymmetric limb extension (2 points); minimal movement of one limb (1 point); and hemiplegia or no limb movement (0 points). The test was repeated three times and the average of the three tests was calculated as previously described (19).

### 2.6 Quantitative Analysis of Microflora

Real-time fluorescence-based quantitative PCR (RT-qPCR) was used to determine the bacterial load. Lung tissues were isolated from mice in the ICH and sham groups removed, cleaned with sterile PBS, weighed, immediately frozen in liquid nitrogen and stored at −80°C until DNA isolation was performed. Total DNA was isolated using an Easy Pure Genomic DNA Kit (TransGen Biotech, China). The RT-qPCR primers used targeted the 16S rRNA-encoding gene and the mouse RNase P/MRP 30 subunit gene (Rpp30). The 16S rRNA gene was amplified using 2.5 µM each primer and 100 ng DNA in a 1 µL reaction, and the Rpp30 gene was amplified with 5 µM each primer and10 ng DNA samples in a1 µL reaction. According to the literature, the 16S rRNA/Rpp30 value was calculated using the 2^−ΔΔCq^ method to represent the bacterial load in lung tissue (13, 20).

### 2.7 RNA Extraction and Quantitative Real-Time PCR

Total RNA was extracted from tissue using an RNA extraction kit (Promega, China). Reverse transcription and quantitative PCR were conducted using the Two-step Bestar qPCR RT Kit and a CFX96 real-time PCR system (Bio-Rad, America) according to the manufacturer’s protocols. The primer sequences are shown in **Table 1**. The relative expression level of the target gene was determined by the 2^−ΔΔCq^ method, and GAPDH was utilized as an internal control. The RT-qPCR primers are listed in **Table 1**.

**Table 1:** Sequences of RT-qPCR primers.

Gene	Primer	Primer sequence (5’-3’)
16S rRNA-encoding gene	Forward	GACTCCTACGGGAGGCAGCAG
	Reverse	GCTGCTGGCACGTAGTTAGCCG
Rpp30	Forward	AGATTTGGATTTAAGAGCG
	Reverse	GAGCAGCAGTCTCCACGAGT
IL-1β	Forward	CCAGGATGAGGACATGAGCA
	Reverse	CGGAGCCTGTAGTGCAGTTG
IL-6	Forward	AGACTTCCATCCAGTTGCCTTCTTG
	Reverse	CATGTGTAATTAAGCCTCCGACTTGTG
TNF-α	Forward	GCGACGTGGAACTGGCAGAAG
	Reverse	GCCACAAGCAGGAATGAGAAGAGG
MCP-1	Forward	CCACTCACCTGCTGCTACTCATTC
	Reverse	CTGCTGCTGGTGATCCTCTTGTAG
MIP-1α	Forward	ACCATGACACTCTGCAACCAAGTC
	Reverse	GCGTGGAATCTTCCGGCTGTAG
MUC2	Forward	TGCTGACGAGTGGTTGGTGAATG
	Reverse	TGATGAGGTGGCAGACAGGAGAC
ZO-1	Forward	AGCTGCCTCGAACCTCTACTCTAC
	Reverse	GCCTGGTGGTGGAACTTGCTC
Occludin	Forward	TGGCTATGGAGGCGGCTATGG
	Reverse	AAGGAAGCGATGAAGCAGAAGGC
Claudin-5	Forward	TGGTGCTGTGTCTGGTAGGATGG
	Reverse	GTCACGATGTTGTGGTCCAGGAAG
GAPDH	Forward	AAGAAGGTGGTGAAGCAGGCATC
	Reverse	CGGCATCGAAGGTGGAAGAGTG

### 2.8 Histological Analysis

At 1, 3, and 7 d after ICH, lung and ileum segments were collected, placed in4% paraformaldehyde, embedded in paraffin and sectioned. The 4-μm-thick sections were stained with hematoxylin and eosin (H&E) and examined under a light microscope (Lecia). Ileum sections were collected and stained with periodic acid-Schiff (PAS) according to standard protocols. For each sample, 200×ileum images were taken using a microscope camera (Lecia).

### 2.9 Measurement of the Spleen and Thymus Indices

The spleens weight was measured immediately after the mice were euthanized. The spleen and thymus indices were calculated as the ratio of spleen or thymus weight (mg) to body weight (g) (21).

### 2.10 Routine Blood Testing

Peripheral blood (50 µL) was collected *via* the orbital vein into a tube containing 100 µL of 10 mM EDTA from mice at 1 d, 3 d, and 7 d after stroke or from those in the sham group and analyzed with a blood cell analyzer (Mairi, BC-2800 Vet).

### 2.11 Measurement of Intestinal Permeability

Intestinal permeability was quantified using the lactulose/mannitol (L/M) test. Twelve hours before the animals in each group were sacrificed, the mouse bladder was emptied, and the animals were given 0.5 mL of the test solution by gastric tube feeding; the test solution contained 60 mg lactulose and 40 mg mannitol. All urine was collected for 12 h through the metabolic cage and stored at -80°C for further analysis. Urinary lactulose and mannitol were measured using high-performance liquid chromatography (Agilent Technologies, USA). The results are presented as a ratio of administered dose of the two.

### 2.12 Measurement of Secretory Immunoglobulin A (SigA) Content

According to the manufacturer’s protocol, the small intestine mucus levels of SigA were measured using commercial enzyme-linked immunosorbent assay (ELISA) kits (Hcusabio Industrial, China). The SigA concentration was calculated using standard curves constructed with serial dilutions of the SigA standard provided within the kit.

### 2.13 Measurement of Lung and Gut Microbiota

Mouse lung and ileum tissues were collected on an ultraclean workbench and stored at −80°C for further use. Microbial DNA was extracted from the samples using the sodium dodecyl sulfate (SDS) method, and DNA purity and concentration were assessed by agarose gel electrophoresis. The 16S V4rRNA gene was sequenced using the 806R (5′-GGACTACHVGGGTWTCTAAT-3′) and 515F (5′-GTGCCAGCMGCCGCGGTAA-3′) primers in lung and ileal tissue samples from mice in the ICH and sham groups. A TruSeq DNA PCR-free Sample Preparation Kit was used to construct the library. High-throughput sequencing was performed on the Illumina MiSeq platform (Illumina, San Diego, CA) following the method detailed by Fadrosh et al. (22).

To study the species composition of each sample, effective tags from all samples were clustered by operational taxonomic unit (OTU) with 97% identity using Uparse v7.0.1001 (http://www.drive5.com/uparse/) (23). We used QIIME software (version 1.9.1) to calculate the Shannon diversity index (24) and R software (version 2.15.3) to draw dilution curves for the alpha diversity index analysis of differences between groups. Beta diversity analysis was performed to indicate differences between groups in relative abundance in lung and gut microbiota. LEfSe software was used for linear discriminant analysis effect size (LEfSe), and the default screening value for the linear discriminant analysis (LDA) score was set to 4, which can be used to compare two or more groups. The LDA score emphasizes statistical significance and biological correlation and can be used to identify significantly different biomarkers between groups. LEfSe was used to analyze differences between groups, and the screening value was set to 4 to identify biomarkers with significant differences among groups.

### 2.14 Statistical Analysis

The data are presented as the mean ± standard deviation (SD) of at least three replicates. To examine differences between two groups, Student’s t-test was conducted. Differences among three or more groups were compared using ANOVA (*P ≤ 0.05*). All analyses were conducted in GraphPad Prism 8 (San Diego, CA, USA).

## 3 Results

### 3.1 The Stroke Model Was Successfully Established, and the Neuromotor Function of Mice Decreased After Stroke

**Figure 1:**
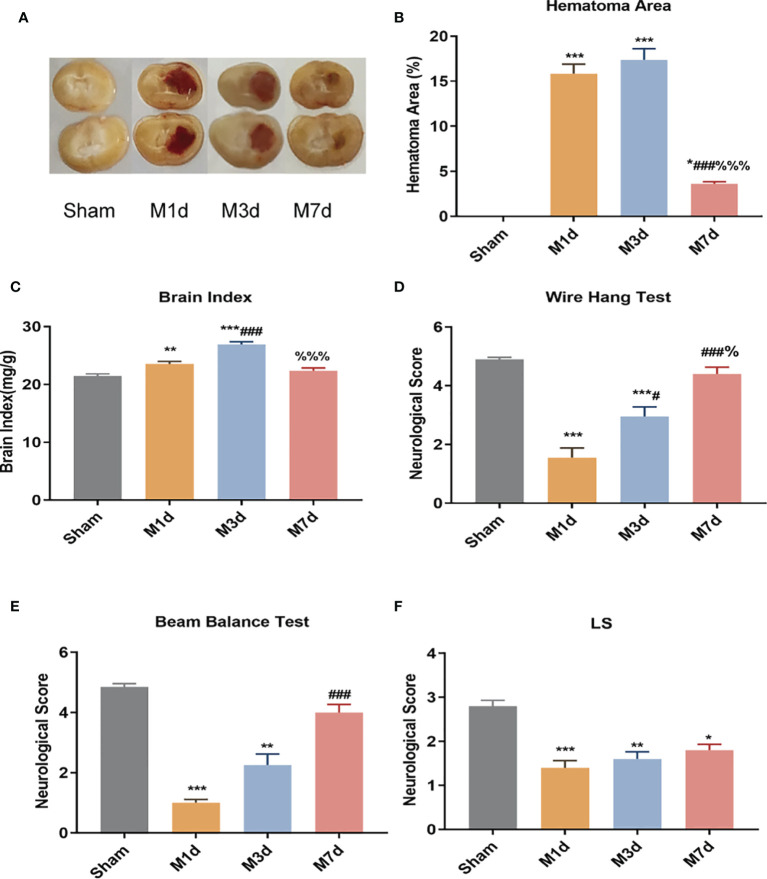
Changes in hematoma area and behavioral scores in the ICH model at different time points. **(A)** Mouse brain slices: the red in the brain tissue is blood. **(B)** Cerebral hemorrhage area ratio (n=5). **(C)** Changes in the brain index in the ICH model (n = 10). ICH neurobehavioral scores at different time points: wire hang test **(D)**, beam balance test **(E)**, and with symmetry of limb movement (Ls) test **(F)** (n = 10). Data are expressed as the mean ± standard deviation. **(B, E)** One-way ANOVA, Tukey’s multiple comparison test; **(C, D)** Brown-Forsythe test and Welch’s ANOVA, Dunnett’s T3 multiple comparison test; **(E)** Kruskal-Wallis ANOVA, Dunnett’s T3 multiple comparison test; *P<0.05, **P<0.01, ***P<0.001 *vs.* the sham group; ^#^ *P*<0.05, ^###^ *P*<0.001 *vs.* the M 1 d group; ^%^ *P*<0.05 *vs.*, ^%%%^ *P*<0.001 the M 3 d group.

The cerebral hemorrhage model of mice was induced by 0.03 U collagenase. The brain sections showed evident bleeding in brain tissue sections of the model (M) group. In contrast, cerebral tissue from mice in the sham group had no bleeding (**Figure 1A**). The cerebral hemorrhage area was significantly larger in the M 1 d, M 3 d and M 7 d groups after stroke than in the sham group (**Figure 1B**) and was significantly smaller in the M 7 d group than in the M 1 d and M 3 d groups. Brain edema was assessed by measuring the brain index. Compared with that in the sham group, the brain index in the M 1 d and M 3 d groups was significantly increased (**Figure 1C**). However, the brain index was significantly decreased at 7 d. The wire hang, beam balance, and LS tests were performed before sampling to indicate the neuromotor function of the mice. Mice in the M 1 d and M 3 d groups had significantly lower wire hang and beam balance test scores than those in the sham group (**Figures 1D, E**). Furthermore, the wire hang test behavioral scores were higher at 3 and 7 d after stroke than 1 d after stroke (**Figure 1D**), and the beam balance test scores were higher in the M 7 d group than the M1 d group (**Figure 1E**). The LS test scores of the sham group were higher than the M 1 d, M 3 d, and M 7 d groups (**Figure 1F**), but were not different among the model groups. The above data showed that one and three days after ICH, the hemorrhage area was large, brain edema was obvious, and neurological function was seriously damaged. Compared with the M 1 d and M 3 d groups, the M 7 d group had significantly decreased bleeding area and brain edema, and increased behavioral scores, suggesting that the brain injury showed signs of recovery in mice at seven days after ICH.

### 3.2 Stroke Induced an Increased Bacterial Load and Immune Disturbance in Lung Tissue on the 7th Day

**Figure 2:**
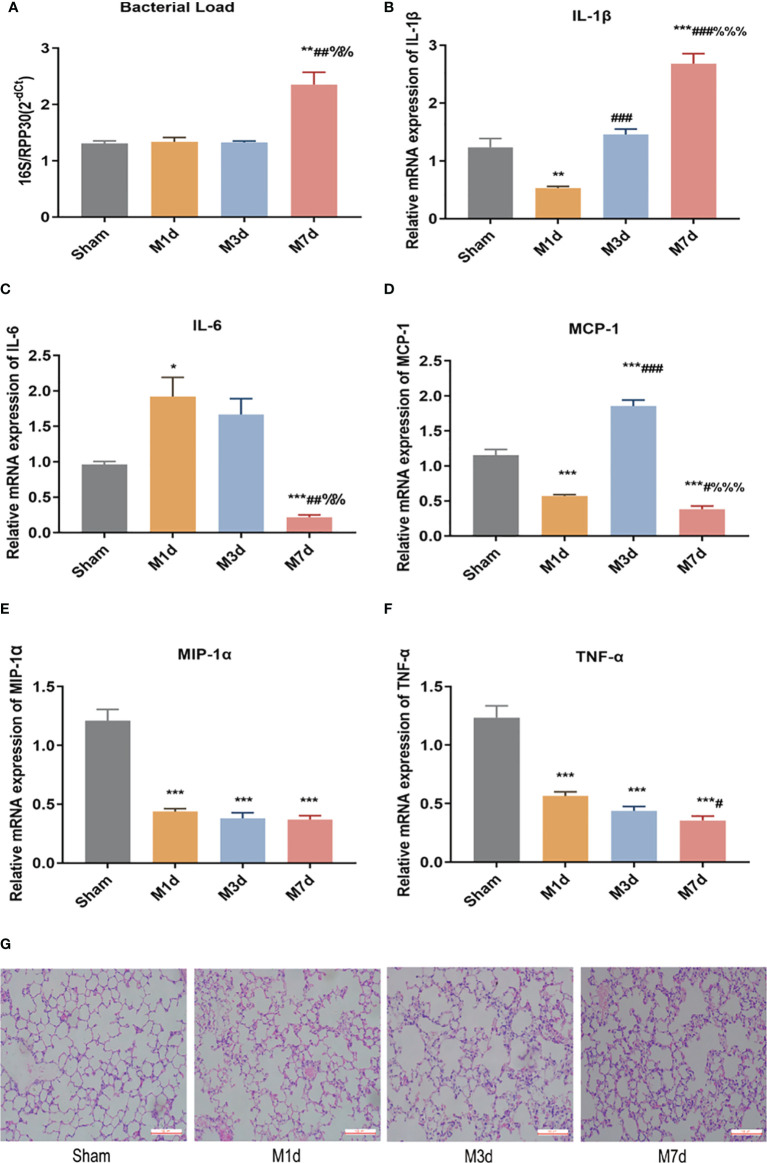
Changes in bacterial load and inflammatory factor expression in lung tissues at different time points in the ICH model. **(A)** Real-time quantitative PCR was used to detect the lung microflora load at different time points in the ICH model (n = 10). **(B–F)** The mRNA levels of IL-1β, IL-6, MCP-1, MIP-1α, and TNF-α in lung tissue from ICH model at different time points were detected by qPCR (n = 8). **(G)** Representative images of H&E staining of the lung at different time points in the ICH model (magnification ×200). Data are expressed as the mean ± standard deviation. **(A, C, D, F)** Brown-Forsythe and Welch ANOVA, Dunnett’s T3 multiple comparison test; **(B, E)** One-way ANOVA, Tukey’s multiple comparison test; *P<0.05, **P<0.01, ***P<0.001 *vs.* the sham group; ^#^ *P*<0.05, ^##^ *P*<0.01, ^###^ *P*<0.001 *vs.* the M 1 d group; ^%%^ *P*<0.01, ^%%%^ *P*<0.001 *vs.* the M 3 d group.

RT-qPCR was used to determine the relative level of total bacteria in lung tissue. The results showed that the bacterial load of lung tissues in the M 1 d and M 3 d groups was not significantly different from that in the sham group. In contrast, the bacterial load of lung tissue in the M 7 d group significantly increased (**Figure 2A**). ICH mice showed lung tissue immune disorders at different time points after stroke. We used qPCR to detect the mRNA levels of IL-1β, IL-6, MCP-1, MIP-1α and TNF-α in lung tissues at different time points after ICH. The results showed that the level of IL-1β in the lung tissue significantly decreased in the M 1 d group (**Figure 2B**), while it was restored to the sham level in the M 3 d group and significantly exceeded this level in the M 7 d group. The level of IL-6 significantly increased in the M 1 d group (**Figure 2C**) but decreased in the M 7 d group. The expression of MCP-1 in lung tissue significantly decreased in the M 1 d and M 7 d groups (**Figure 2D**) but increased dramatically in the M 3 d group. MCP-1 expression in the M 7 d group was significantly lower than that in the M 1 d and M 3 d groups, MIP-1α and TNF-α levels decreased in the M 1 d, M 3 d and M 7 d groups compared with the sham group (**Figures 2E, F** **)**. Furthermore, the level of TNF-α on the seventh day poststroke was an order of magnitude lower than that on the first day after ICH. Our results indicated that experimental stroke caused an increase in the number of bacteria in lung tissue, immune disorder and a persistent inflammatory response. On the seventh day after ICH, the levels of most inflammatory factors decreased in lung tissue, which showed immunosuppression. The lung histopathology results revealed that normal structure of alveolar wall in the sham group, and no inflammatory cell infiltration in the alveolar septum (**Figure 2G**). In the M 1 d, M 3 d and M 7 d groups, the alveoli were dilated, the alveolar septum was thickened, edema and inflammatory cell infiltration were present, and protein leakage in the alveoli was occasionally observed; lung damage worsened over time. The lung tissue lesions in the M 7 d group were the severest, which may be related to the lung tissue immune disorder and bacterial infection.

### 3.3 The Peripheral Immune Response Was Dysregulated in Mice Following Stroke

**Figure 3:**
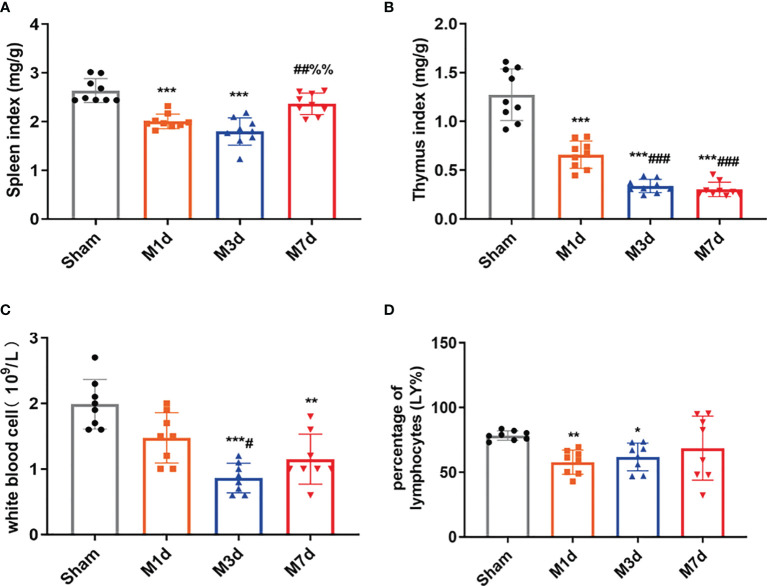
Changes in immune function in the ICH model at different time points. **(A, B)** Changes in the spleen and thymus indices in ICH mice (n = 9). **(C, D)** WBC count and lymphocyte percentage (LY%) in peripheral blood were detected by a routine blood analyzer (n = 8). Data are expressed as the mean ± standard deviation. **(A–D)** Brown-Forsythe test and Welch’s ANOVA, Dunnett’s T3 multiple comparison test; *P<0.05, **P<0.01, ***P<0.001 *vs.* the sham group; ^#^ *P*<0.05, ^##^ *P*<0.01, ^###^ *P*<0.001 *vs.* the M 1 d group; ^%%^ *P*<0.01 *vs.* the M 3 d group.

In several acute neurological conditions, decreased immune competence with a higher incidence of infection has been demonstrated (25). The thymus and spleen are critical lymphatic organs that participate in immune regulation, and changes in thymus and spleen quality are important signs of changes in the immune environment. The spleen and thymus indices can partly reflect immune activity in the body. In this study, the splenic and thymus indices were selected to initially observe changes in lymphatic organs after ICH. The experimental results showed that the spleen index significantly decreased in the M 1 d and M 3 d groups (**Figure 3A**), but it was restored to the sham level on day 7 poststroke. However, the thymus index did not show the same trend as the spleen index. The thymus index significantly decreased in the M 1 d, M 3 d, and M 7 d groups (**Figure 3B**), with significant reductions in the M 3 d and M 7 d groups compared with the M 1 d group. The routine blood test results showed that the white blood cell (WBC) count significantly decreased in the M 3 d and M 7 d groups after ICH (**Figure 3C**), and the lymphocyte percentage (LY%) showed a decreasing trend in the M 1 d and M 3 d groups (**Figure 3D**). The results indicated that immune function was disrupted in the M 1 d, M 3 d, and M 7 d groups, with the most severe suppression in the M 3 d group, but a partial recovery in the M 7 d group.

### 3.4 Intestinal Barrier Dysfunction Was Aggravated Over Time After Stroke

**Figure 4:**
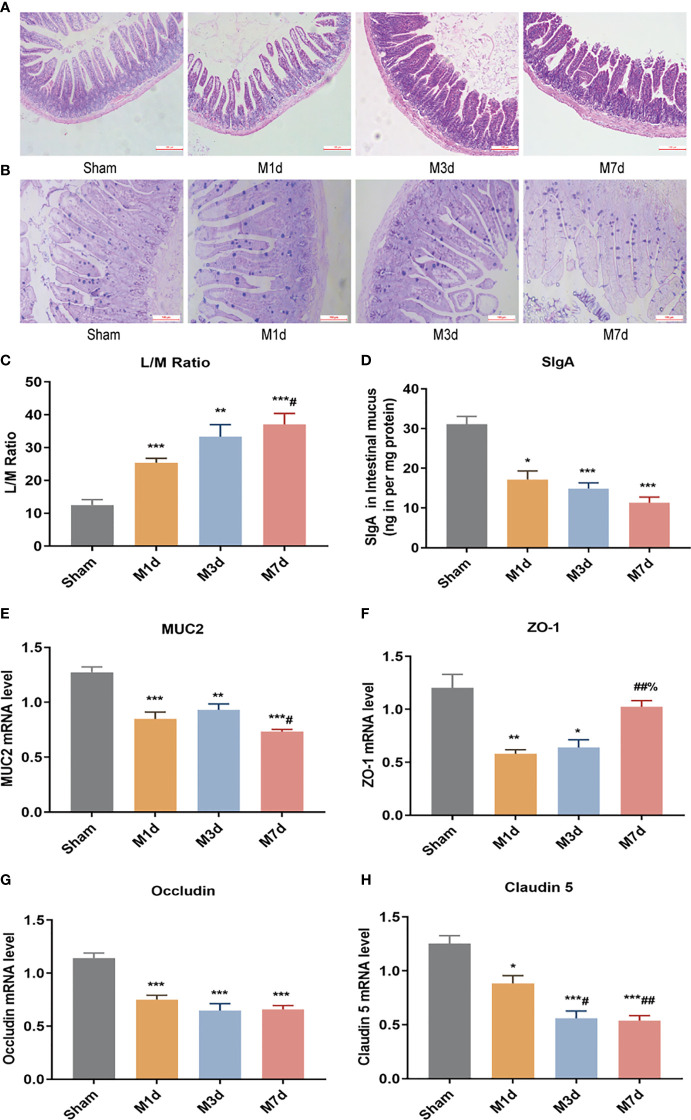
Changes in intestinal barrier function in the ICH model at different time points. **(A)** Representative images of H&E staining of the ileum at different time points in the ICH model (magnification ×100). **(B)** Representative images of PAS staining of the ileum at different time points in the ICH model (magnification ×200). **(C)** L/M ratio, indicating changes in intestinal permeability at 1, 3 and 7 d following ICH (n=8). **(D)** Small intestine SigA level was quantified by ELISA at 1, 3 and 7 d following ICH (n=8). **(E, F)** The mRNA levels of MUC2, ZO-1, Occludin, and Claudin-5 were measured with RT-qPCR and normalized to GAPDH mRNA level (n = 8). Data are expressed as the mean ± standard deviation. **(C)** One-way ANOVA, Tukey’s multiple comparison test; **(D, E)** Brown-Forsythe test and Welch’s ANOVA, Dunnett’s T3 multiple comparison test; **(F)** Kruskal-Wallis ANOVA, Dunn’s multiple comparison test; **(G, H)** One-way ANOVA, Dunnett’s T3 multiple comparison test; *P<0.05, **P<0.01, ***P<0.001 *vs.* the sham group; ^#^ *P*<0.05, ^##^ *P*<0.01 *vs.* the M 1 d group; ^%^ *P*<0.05 *vs.* the M 3 d group.

The intestinal epithelial barrier is a critical component of defense mechanisms required to prevent infection. HE and AB-PAS staining of ileum tissue from the sham group (**Figures 4A, B**) showed that the ileum terminal mucosa epithelium was intact, with normal villi and a well-defined arrangement of cup cells and intestinal glands. After ICH, the villi of the small intestine were exfoliated, the striatal border was damaged, the upper and proper layers were blurred, the intestinal glands were smaller, inflammatory cell infiltration of the submucosa was obvious, and fewer cup cells were present. The lesions in the M 3 d group were worse than those in the M 1 d group, whereas the lesions were similar in the M 3 d and M 7 d groups. In this experiment, intestinal permeability was evaluated by detecting the L/M leakage rate in the urine of mice in each group. The results showed that compared with the sham group, the M 1 d, M 3 d and M 7 d groups showed a trend of increasing intestinal permeability over time, with the greatest intestinal permeability in the M 7 d group (**Figure 4C**). SigA significantly decreased in the M 1 d, M 3 d and M 7 d groups (**Figure 4D**). Goblet cells produce MUC2 to protect the intestinal tract from self-digestion and numerous microorganisms (26). Compared with that in the sham group, the mRNA transcript level of MUC2 in the M 1 d, M 3 d and M 7 d groups significantly decreased (**Figure 4E**). In addition, MUC2 mRNA level significantly lower in the M 7 d group than in the M 1 d group. The integrity of the intestinal barrier is maintained predominantly by tight junctions (TJs) between intestinal epithelial cells (IECs) (27). qPCR was used to detect changes in the mRNA expression of the TJ genes ZO-1, Occludin, and Claudin-5 at different times after ICH (**Figures 4F–H**). ZO-1 mRNA level significantly decreased in the M 1 d and M 3 d groups compared with the sham group (**Figure 4F**), but there was no significant difference in Claudin-5 expression. In contrast, ZO-1 mRNA expression markedly increased in the M 7 d group compared to the M 1 d and M 3 d groups. Occludin mRNA level significantly decreased in the M 1 d, M 3 d and M 7 d groups (**Figure 4G**). Claudin-5 mRNA level in lung tissue decreased in the M 1 d, M 3 d and M 7 d groups compared to the sham group (**Figure 4H**). Moreover, Claudin-5 level was significantly lower in the M 3 d and M 7 d groups than in the M 1 d group. These results suggest that intestinal barrier damage occurs in the early stage of ICH. Intestinal barrier function does not recover over time after stroke, which may lead to the translocation of harmful microorganisms.

### 3.5 Stroke-Induced Intestinal Bacterial Translocation to the Lung on Day 7

**Figure 5:**
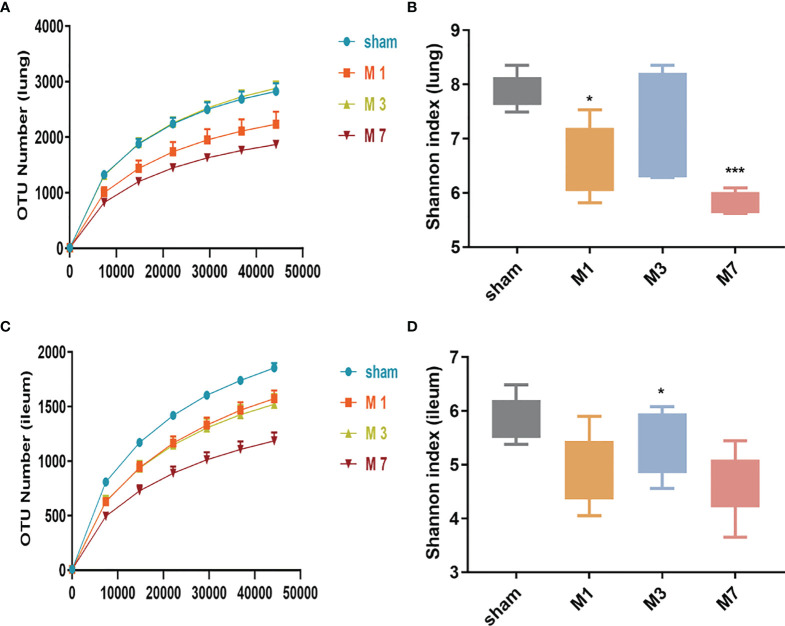
Species richness and diversity of the gut microbiome in the ICH model at different time points. **(A, B)** The rarefaction curve and Shannon diversity index in the lung show significantly reduced species diversity on days 1 and 7 after ICH. **(C, D)** The rarefaction curve and Shannon diversity index in the ileum show significantly reduced species diversity induced on day 7 d after ICH. Data are expressed as the mean ± standard deviation. *P<0.05 and ***P<0.001. *vs.* sham group.

In this study, high-throughput sequencing analysis was used to compare changes in bacterial community structure in lung and intestinal tissues after ICH. Dilution curve analysis showed that the sequencing depth was sufficient to reflect most of the microbial diversity information in the samples (**Figures 5A, C** **)**. Compared with the sham group, the M 1 d and M 7 d groups showed a significant reduction in the α diversity of the microbiota in lung tissue (**Figure 5B**). The α diversity of the intestinal microflora decreased on the first, third and seventh days after experimental stroke, and the species α diversity significantly reduced on the seventh day after stroke (**Figure 5D**).

**Figure 6:**
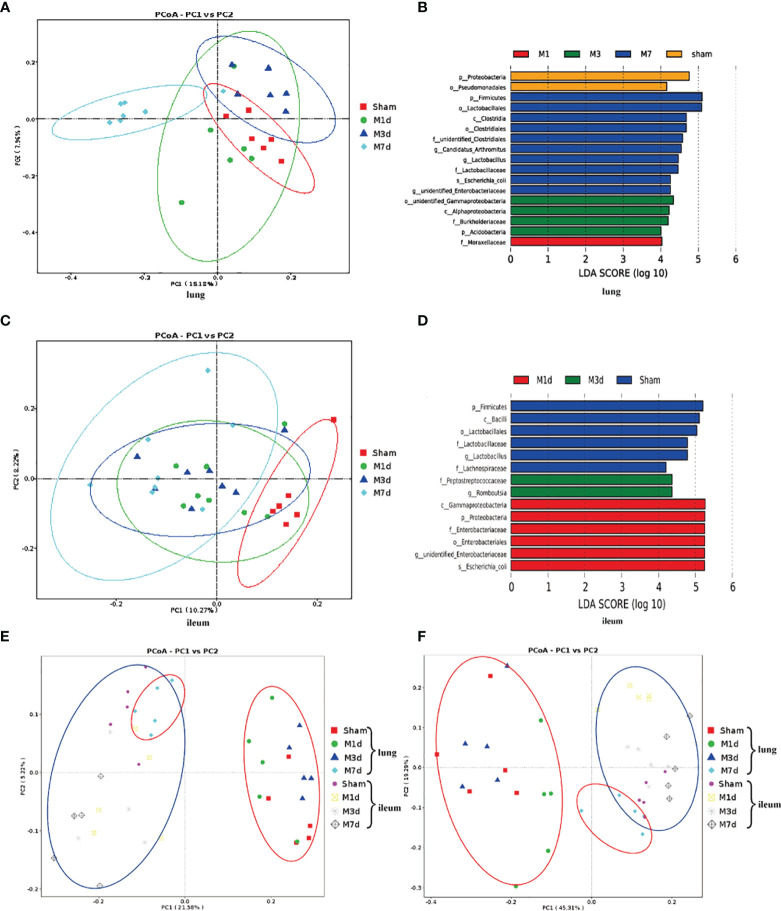
Differences in the lung and ileum microbiota of the ICH model at 1 d, 3 d and 7 d. **(A)** The microbial communities in lung samples in the ICH model at different time points were compared using a PCoA plot based on a weighted UniFrac distance metric. **(B)** The LEfSe method was used to analyze the differences in bacterial flora among groups and to identify the specific bacterial flora in lung tissue from each group (LDA score > 4). **(C)** The microbial communities in ileum samples at 1 d, 3 d and 7 d following ICH were compared using a PCoA plot based on a weighted UniFrac distance metric. **(D)** The distribution of LDA values in ileum tissue displayed as a histogram, with an LDA value of >4 indicating a statistically significance among groups. **(E, F)** We next used unweighted and weighted UniFrac analyses to explore the relationship between lung microbiota samples and ileum samples collected at 1, 3 and 7 d following ICH (red ovals, lung tissue samples; blue ovals, ileum tissue samples).

We further analyzed the changes in the microbiome composition of different lung and intestinal tissue samples over time. Principal coordinates analysis (PCoA) of lung tissue samples showed that the community composition was significantly different in the M 7 d and sham groups (**Figure 6A**). Linear discriminant analysis effect size (LEfSe) was used to find biomarkers with a significant difference among groups (**Figure 6B**). We confirmed that the phylum *Proteobacteria* and the order *Pseudomonadales* were more prevalent in the lung microbiota from the sham group. The most common flora in lung tissue from the M 1 d group was in the family *Moraxellaceae*. The lung microbiota in the M 3 d group included higher representation of the phylum *Acidobacteria*, the classes *Alphaproteobacteria* and unidentified *Gammaproteobacteria*, and the family *Burkholderiaceae*. In contrast, the lung microbiota from the M 7 d group had higher levels of the phylum *Firmicutes*; the class *Clostridia*; the orders *Clostridiales* and *Lactobacillales*; the families unidentified *Clostridiales* and *Lactobacillaceae*; the genera *Lactobacillus*, *Candidatus Arthromitus*, unidentified *Enterobacteriaceae*; and the species *Escherichia coli*. The PCoA results of intestinal tissues showed that in the unweighted UniFrac PCoA diagram, only the M 7 d group was completely distinguished from the sham group (**Figure 6C**). The LEfSe analysis results of intestinal tissues showed (**Figure 6D**) that the sham group’s enriched flora were the phylum *Firmicutes*, the order *Lactobacillales*, the families *Lactobacillaceae* and *Lachnospiraceae*, and the genus *Bacilli*. The phylum *Proteobacteria*, the class *Gammaproteobacteria*, the order *Enterobacteriales*, the family *Enterobacteriaceae*, and the species *Escherichia coli* were higher in the gut microbiota in the M 1 d group. Compared with the other groups, the M 3 d group had greater abundance of the family *Peptostreptococcaceae* and genus *Romboutsia* in the gut microbiota.

PCoA was used to reduce dimensions and display the differences among samples. The PCoA results of lung and intestinal samples showed that in the unweighted and weighted UniFrac analyses (**Figures 6E, F** **)**, the microflora structure of lung tissue in the ICH model on the seventh day was distinguished from that in the ICH model on days 1 and 3 but was similar to that in the intestinal tissue, suggesting that lung tissue infection may be related to the intestinal microflora.

## 4 Discussion

One of the most commonly used ICH models is the collagenase injection model, firstly established by Rosenberg in 1990 (28). Upon injection of bacterial collagenase, the basement membrane of small blood vessels dissolves and in turn causes cerebral parenchyma hemorrhage that lasts several hours. This model is stable during surgery and can be used to study neurological rehabilitation after acute hemorrhage and ICH. Several studies found a strong association between poor neurological function and poststroke infections in patients with acute stroke. Stroke severity is an essential predictor of poststroke infection. Our study indirectly assessed the likelihood of secondary lung infection after stroke with the neurological function score. In our study, the area of brain hematoma, degree of brain edema, and severity of nerve injury were greater in the M 1 d and M 3 d groups after hemorrhagic stroke than in the M 7 d and sham groups, an indicative of recovery for the brain injury of mice on day 7 poststroke.

The lungs of patients with severe brain injury, including that related to ischemic and hemorrhagic strokes, are particularly vulnerable (29). *Bai et al.* reported that 15.6% of stroke patients developed acute lung injury within 36 h of hospitalization, and 7.8% developed pneumonia or bronchitis during hospitalization (30). Studies have shown that ischemic stroke causes excess WBCs to infiltrate the lungs, and reduces the phagocytic capability of alveolar macrophages (31). Excessive leukocyte infiltration into the lungs leads to the release of various toxic factors that aggravate lung injury. Inhibiting abnormal pneumonic responses has been shown to protect against lung damage (32). We conducted an exploratory study on the mechanism of secondary pulmonary infection after ICH. In particular, we assessed the changes in inflammatory factors and the pathological structure of lung tissues on the first, third and seventh day after ICH in mice. H&E staining showed that the lung lesions in ICH mice were most serious on the seventh day. Cytokines are essential effectors and regulators of the immune system. IL-1β, IL-6, TNF-α, MIP-1α, and MCP-1 are inflammatory factors secreted by monocytes/macrophages.MIP-1α and MCP-1 are chemotactic cytokines that play an important role in promoting the circulation of effector cells to inflammatory sites (33, 34). Our results showed that the expression of inflammatory factors and chemokines in lung tissue was altered at different time points in model mice (**Figure 2**), indicating immunologic disorder in lung tissue after ICH. As stroke results in disordered cytokine homeostasis, it is likely that stroke-induced immunosuppression, in combination with exacerbated lung lesions, permits the translocation of bacteria to the lung.

Early brain injury leads to activation followed by suppression of the immune system (35). However, excessive suppression of the immune system during brain injury can increase the probability of infection, thus affecting the recovery and mortality of stroke patients and laboratory animals (36, 37). Systemic inflammation peaks within 6 h after stroke (38). To control excessive inflammatory and immune responses, systemic immune suppression occurs as a compensatory mechanism to avoid brain injury. Previous studies have shown that different does of collagenase induce brain hematomas of varying size and different spleen weights/areas; in the ICH model, the spleen weight varies at different time points (39). Considering the changes in hematoma size and immune function, we measured lymphoid organ indices at 1, 3, and 7 d in mice treated with the 0.03 U collagenase to induce ICH Compared with those in the sham group, mice in the M 1 d, M 3 d and M 7 d groups exhibited significant thymus and spleen atrophy, with the most significant atrophy in the M 3 d group and slight recovery in the M 7 d group. Total WBC and lymphocyte counts can be used to predict infection after stroke (40, 41). The total numbers of circulating leukocytes and lymphocytes were reported to decrease after stroke in both animal models and human subjects (42, 43). We found fewer leukocytes and lymphocytes in the peripheral blood poststroke, with the most dramatic decrease occurring on day 3. We speculated that this finding may indicate that diminished peripheral immune defense increases the risk of poststroke infection. Stroke injury may first activate the hypothalamic pituitary adrenal (HPA) axis through glucocorticoids, which induce peripheral splenic atrophy and then cause lymphopoiesis defects and NK cell deficiency, further leading to peripheral immunosuppression. The impaired immune system reduced the host defense against bacteria and increased host susceptibility.

The intestinal epithelial barrier is formed by several highly integrated physical (the epithelium), biochemical (mucus, antimicrobial peptides, and AMP) and immunological (SigA) elements (44). Brain damage can lead to damage to the intestinal mucosa, increased intestinal inflammation and intestinal dysfunction (41, 45). We observed persistent ileal mucosal damage in ICH mice with reduced goblet cell numbers. ICH may result in a breakdown of communication between the gastrointestinal system and central nervous system, resulting in gastrointestinal motility disorders and permeability. We found increased intestinal permeability and decreased TJ protein expression following stroke. On the 7th day poststroke, intestinal permeability was the highest, accompanied by the mRNA transcript levels of the TJ proteins Occludin and Claudin-5 were decreased. These results suggest that stroke disrupts the intestinal mechanical barrier in mice. Intestinal SigA is an important component of the intestinal mucosal immune barrier, and MUC2 is a major component of the intestinal mucus layer (34, 46, 47). It has been reported that alterations in intestinal MUC2 and microbial diversity are closely linked with critical intestinal pathologies (48). The expression of intestinal SigA and MUC2 in mice was significantly decreased in poststroke. Moreover, increased intestinal permeability and decreased expression of TJ proteins, mucin, and immunoglobulin after stroke lead to impaired intestinal barrier function, making it easier for gut bacteria enter surrounding tissues and organs, thus leading to systemic infection.

The brain-gut axis is a two-way communication system between the central nervous system and gut (49). Under normal physiological conditions, the intestinal flora participates in regulating host immunity, nutrient absorption, and intestinal movement to maintain homeostasis (50). In disease states, translocation and transformation of gut microbes may have significant pathological consequences for the host. Studies have shown that stroke can cause significant changes in the intestinal microbiota, leading to an overall reduction in the species diversity of the intestinal microbiota (51). Antibiotic intervention can reduce the severity of cerebral infarction after ischemic stroke (52). Our study found that ICH led to changes in the lung and ileum microflora diversity at the first, third and seventh days after stroke; the structure of the lung microflora was similar to that of the intestinal microflora on day 7 poststroke, which was consistent with the significant increase in lung microflora at this time point (**Figures 2**, **5**). Alterations in gut microbial composition increase susceptibility to respiratory diseases and promote bacterial migration, which may induce poststroke infections (15, 53). Types of bacteria that have been commonly detected in the sputum and urine of stroke patients are common commensal bacteria that reside in the human intestinal tract (e.g., *Enterococcus* spp. and *Escherichia coli*) *(* 13, 54–57). This finding suggests that spontaneous pulmonary infection caused by stroke may be derived from transposed host gut microbiota.

Our study has several limitations. First, the method of generating ICH models or the choice of collagenase dosage may affect the model performance. We used a model of collagenase injection and cannot rule out the possibility that the results would differ in other models of brain damage. Second, our study preliminarily evaluated the influence of stroke at different time points on immune function without lymphocyte typing, but this is a line of future investigation in the laboratory. Third, although we described a vital source of secondary infection poststroke, we did not establish bacterial translocation and dissemination after the experimental induction of stroke, which will be addressed in a future study.

In summary, our results suggest that hemorrhagic stroke induces significant damage to the immune system and disruption of barrier function; this damage occurs rapidly, as early as the first day following hemorrhagic stroke, and exacerbated over time. These alterations increase susceptibility to poststroke pneumonia, which can develop into a lung infection by the seventh day after hemorrhagic stroke. Thus, early and timely intervention is necessary to reduce the risk of infection-related mortality and to improve the prognosis of pneumonitis poststroke.

## Data Availability Statement

The raw data supporting the conclusions of this article will be made available by the authors, without undue reservation.

## Ethics Statement

The animal study was reviewed and approved by The Animal Research Center of Tianjin University of Traditional Chinese Medicine. Written informed consent was obtained from the owners for the participation of their animals in this study.

## Author Contributions

YZ and PZ designed the study. YH and HZ performed the experiments. YH and HZ contributed equally and should be considered co-first authors. PZ, YH, and HZ analyzed the data. HZ and PZ wrote the manuscript. XL, XH, JH, BW, and LZ performed the laboratory work. All authors contributed to the article and approved the submitted version.

## Funding

This work was supported by the National Natural Science Foundation of China (#82174112).

## Conflict of Interest

The authors declare that the research was conducted in the absence of any commercial or financial relationships that could be construed as a potential conflict of interest.

## Publisher’s Note

All claims expressed in this article are solely those of the authors and do not necessarily represent those of their affiliated organizations, or those of the publisher, the editors and the reviewers. Any product that may be evaluated in this article, or claim that may be made by its manufacturer, is not guaranteed or endorsed by the publisher.
